# A proposed modification to the Kellgren and Lawrence classification for knee osteoarthritis using a compartment‐specific approach

**DOI:** 10.1002/jeo2.12008

**Published:** 2024-02-23

**Authors:** Diego Alarcón Perico, Abelardo Camacho Uribe, Sara Jaimes Niño, María Camila Peñaloza Mayorga, Christian Sundfeld, Jorge Rojas Lievano, Cristal Castellanos Mendoza, Rafael Gómez Ramirez, Oscar Rivero Rapalino, Gamal Zayed, German Carrillo Arango, Klaus Mieth

**Affiliations:** ^1^ Department of Orthopedics and Traumatology Hospital Universitario Fundación Santa Fe de Bogotá Bogotá Colombia; ^2^ Department of Radiology Hospital Universitario Fundación Santa Fe de Bogotá Bogotá Colombia; ^3^ School of Medicine Universidad de Los Andes Bogotá Colombia

**Keywords:** classification, compartment‐specific approach, Kellgren and Lawrence, knee osteoarthritis

## Abstract

**Purpose:**

Since Kellgren and Lawrence (KL) originally classified knee osteoarthritis, several authors have reported varying levels of reliability and a lack of uniformity in the use of this classification system. We propose several modifications to the KL classification including the use of a compartment‐specific approach that we hypothesize will lead to a better understanding of knee OA while maintaining an adequate interobserver and intraobserver reliability.

**Methods:**

We propose the addition of the lateral and skyline‐view radiographs to the standard anteroposterior (AP) and lateral projections in the evaluation. Also suggest a more precise definition of the evaluated parameters; the addition of the subchondral cancellous bone as parameter of evaluation; and the assessment of medial tibiofemoral compartment (MTFC), lateral tibiofemoral compartment (LTFC) and patellofemoral compartment (PFC) separately resulting in a compartment‐specific KL staging score rather than a single overall KL score. Six evaluators (two knee surgeons, two radiologists and two knee fellows) used the modified KL classification to classify 230 randomly selected knees on two separate occasions. Reliabilities were assessed by calculating Krippendorff's *⍺* coefficients.

**Results:**

Two hundred and ten knees were included for final evaluation and analyses (53% left knees; 65% females; mean age 56 years old). Average interobserver reliability was moderate for all compartments (0.51 for the MTFC; 0.51 for the LTFC; and 0.56 for the PFC). Average intraobserver reliability was substantial for all compartments (0.63 for the MTFC; 0.65 for the LTFC; and 0.7 for the PFC). Experienced evaluators showed a higher intraobserver reliability than less‐experienced evaluators.

**Conclusions:**

A modified compartment‐specific KL classification enables a practical and detailed description of knee OA involvement and demonstrates acceptable interobserver and intraobserver reliability.

**Level of Evidence:** Level III

**Abbreviations**
Alpha: Krippendorff's *⍺* coefficient
AP: anteroposterior
ICC: interclass correlation coefficient
Kappa: Cohen's *κ* coefficient
KL: Kellgren and Lawrence
LTFC: lateral tibiofemoral compartment
MTFC: medial tibiofemoral compartment
OA: osteoarthitis
PFC: patellofemoral compartment
PFOA: patellofemoral OA
TF: tibiofemoral
TFOA: tibiofemoral OA

## BACKGROUND

Knee osteoarthritis (OA) is a common condition among patients older than 60 years with a reported prevalence of up to 40% [1, 2]. Plain radiographs are the gold‐standard for establishing the diagnosis as well as for severity and progression evaluation in patients with knee OA [3, 4]. Radiographic findings of knee OA include osteophytes; subchondral sclerosis and cysts; and joint space narrowing [5]. Several classification systems have been proposed for staging knee OA based on the extent of these radiographic findings. The Kellgren and Lawrence (KL) is the most widely used classification system for staging knee OA [3, 4, 6, 7, 8]. Originally described an increasing severity of knee OA using exclusively anteroposterior (AP) knee radiographs using a grading scale from 0 to 4. Grade 0 signifying absence of OA and Grade 4 severe OA (Table 1) [9]. Several studies have reported on the interobserver and intraobserver reliability of the KL classification with varying results that range from poor to substantial agreement [10, 11, 12].

**Table 1:** Original Kellgren and Lawrence classification [9].

Grade	Characteristics
0	No JSN or reactive changes
1	Doubtful JSN, possible osteopythic lipping
2	Definite ostephytes, possible JSN
3	Moderate osteophytes, definite JSN, some sclerosis, possible bone‐end deformity
4	Large osteophytes, marked JSN, severe sclerosis, definite bone ends deformity

*Note*: Modified from Kohn MD, Sasson AA, Fernando ND. Classifications in brief: Kellgren‐Lawrence Classification of Osteoarthritis. Clinical Orthopaedics & Related Research. 2016;474(8); 1886–1893.

Abbreviation: JSN, joint space narrowing.

The original KL classification was limited in several ways. First, its original description was inconsistent by the authors which resulted in variable application of the classification in subsequent studies. In their original paper, KL provided simple descriptions of each grade, “none, doubtful, minimal, moderate, severe,” along with radiographic features considered evidence of OA [13]; however, they never explicitly specified which radiographic features correspond to which grade [9]. Second, it only used the AP knee radiograph for assessment, excluding patellofemoral arthritis as a distinct radiographic factor of knee OA. Third, in contrast to other joints, the knee is composed by multiple compartments that may be affected by OA in diverse degrees which leads to distinct radiographical and clinical OA patterns. The original KL classification does not take this into consideration assessing the joint with an overall score and not by compartment [14]. This may not accurately reflect the extent of involvement in each compartment of the knee joint which might result in disagreements in OA staging between raters and misinterpretations of the actual extent of disease, especially when evaluating patients with early OA limited to a single compartment.

The goal of this study is to propose a modification of the KL classification using a compartment‐specific approach to enable a more practical and reliable description of joint involvement. We hypothesize that this modification to the KL classification will lead to a better understanding of knee OA while maintaining an adequate interobserver and intraobserver reliability.

## MATERIALS AND METHODS

**Figure 1:**
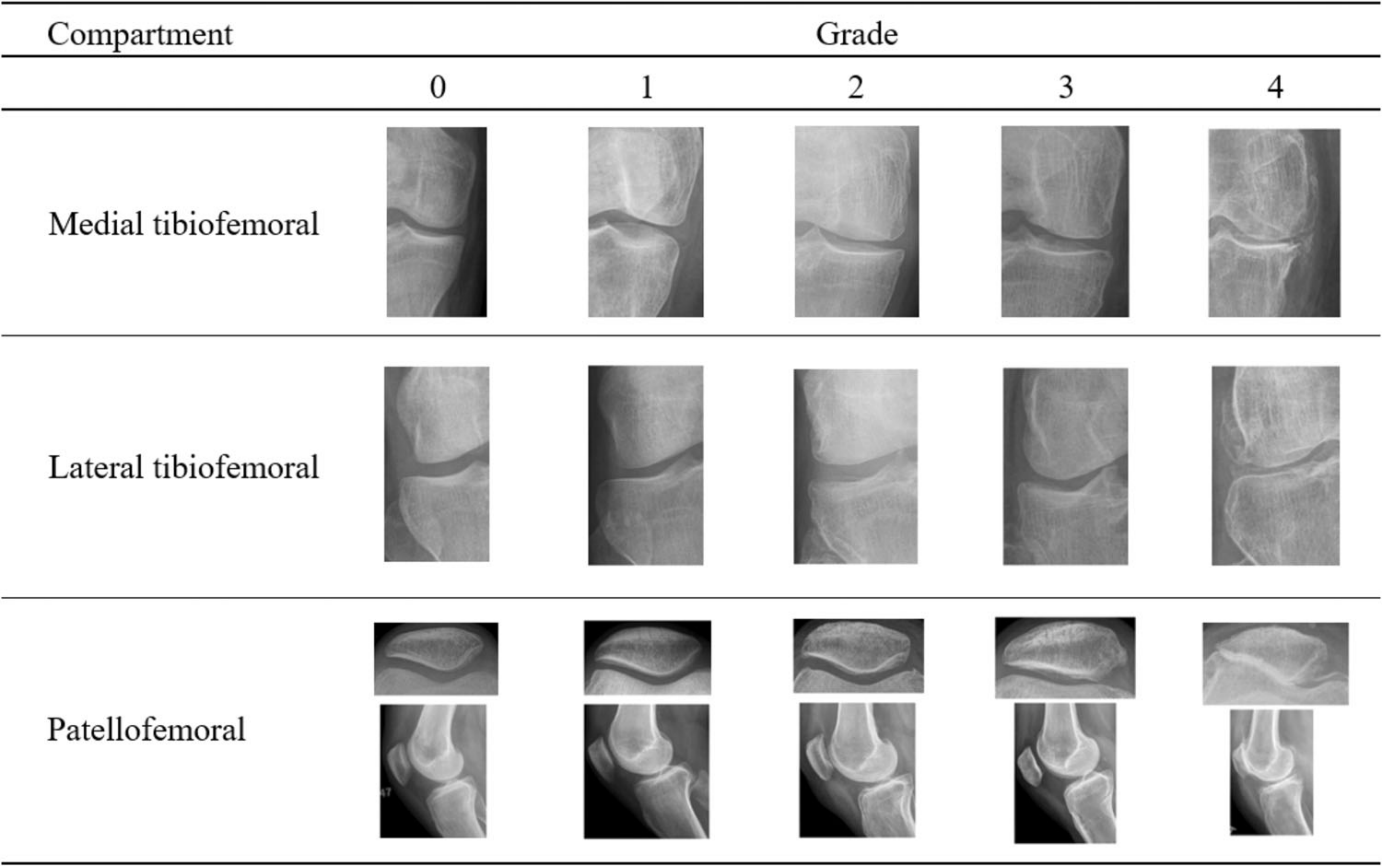
Compartment‐specific stages of Kellgren and Lawrence modified classification.

This cross‐sectional study proposes several modifications to the original KL classification system (Figure 1). These modifications were based on an informal consensus of a group of experts composed by two experienced orthopedic surgeons with fellowship training in knee surgery and two musculoskeletal radiologists with >10 years of experience. Specifically, we suggest: (1) the addition of the lateral and skyline view radiographs in the evaluation; (2) a more precise definition of the evaluated parameters; (3) the addition of the subchondral cancellous bone as parameter of evaluation; and (4) the assessment of each compartment separately in an ordered manner that results in compartment‐specific KL staging score rather than a single overall KL score. The Institutional Review Board and Ethics Committee of the institution approved and monitored the study (CCEI‐8601‐2017).

**Figure 2:**
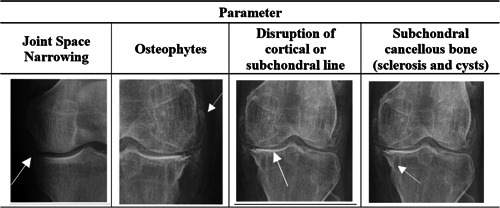
Compartment‐specific parameters of evaluation in the Kellgren and Lawrence modified classification.

We define four parameters of evaluation based on radiographic findings: (1) joint space narrowing, (2) presence of osteophytes (including the tibial spines), (3) disruption of cortical or subchondral line, and (4) subchondral cancellous with presence of bone sclerosis and cysts (Figure 2).

Lastly, we propose the following staging score which is generic for the three knee compartments; since we propose four parameters of evaluation, we outline one parameter whose presence is sufficient to define each stage aiming to improve the differentiation between stages and the agreement between evaluators. We define a Stage 0 compartment as one with normal joint space, no osteophytes, and normal subchondral line and subchondral cancellous bone. The Stage 1 differs from the Stage 0 only by the presence of minimal osteophytes and thus, the sufficient parameter to define a Stage 1 compartment is the presence of minimal osteophytes. The Stage 2 differs from the Stage 1 by the presence of mild joint space narrowing, defined as <50% narrowing. The presence of mild joint narrowing is sufficient to define a Stage 2 compartment. While the subchondral cancellous bone may exhibit sclerosis in Stage 2, this finding is not considered sufficient nor necessary to define a Stage 2 compartment. We define a Stage 3 compartment as one with moderate joint space narrowing, defined as >50% narrowing. Likewise, compromise of the subchondral bone with the presence of subchondral cysts is an indicator of Stage 3. The presence of any of these two parameters is considered sufficient to define a Stage 3 compartment. The presence of subchondral cysts is especially helpful to define a Stage 3 in the PFC due to the limitations to reliably assess joint narrowing in this compartment. Other findings of Stage 3, that are not necessary nor sufficient, include moderate or significant osteophytes, sclerosis of the subchondral bone and irregular subchondral line. The Stage 4 differs from the Stage 3 by the presence of severe joint space narrowing which is close to 100%, significant osteophytes, and depression of the subchondral line (Table 2).

**Table 2:** Modification of Kellgren and Lawrence classification.

Parameter	Grade				
	0 (Normal)	1 (Unclear)	2 (Mild)	3 (Moderate)	4 (Severe)
Joint Space	Normal	Normal	Mild narrowing < 50%^a^	Moderate narrowing > 50%^a^	Narrowing close to 100%^a^
Ostheophytes (Including tibial spines)	None	Minimal^a^	Minimal	Moderate or significant	Significant
Cortical or subchondral line	Normal	Normal	Normal	Irregular	Irregular/Depression^a^
Subchondral cancellous bone	Normal	Normal	Normal or sclerosis	Cyst with or without sclerosis^a^	Cyst with or without sclerosis

a Parameter whose presence is sufficient to define each stage.

The evaluation of the compartments is performed in a systematic and ordered manner starting with the medial tibiofemoral compartment (MTFC), followed by the lateral tibiofemoral compartment (LTFC), and lastly the patellofemoral compartment (PFC). For radiographic evaluation of each compartment, we use the conventional AP and lateral projection for MTFC and LTFC, and lateral and skyline view for PFC.

### Study population

Inclusion criteria were patients 18 years old or older with the following radiographic projections: weight‐bearing AP; lateral with the knee flexed to 30°; and the skyline view with the knee flexed to 45° as proposed by Merchant [15]. All radiographs were taken using the same technique on the same machine by radiology technicians trained in this subject and were evaluated in the same digital system (Xero Viewer; Agfa Healthcare). Exclusion criteria included (1) low‐quality radiographs, (2) incorrect projections, (3) the presence of osteosynthesis or prosthetic material, and (4) history of previous surgery, trauma, or congenital deformities on the index knee. From the potentially eligible cases, included cases were selected following a stratified random sampling by age groups to represent the whole spectrum of knee OA.

The population of evaluators included the initial four participants of the informal consensus and two orthopedic knee fellows, resulting in a total of six evaluators. Before starting the first series of assessments, all the evaluators met to discuss the definitions of the modified classification system. Written detailed descriptions were provided to each evaluator to avoid misunderstandings of the modified classification and each evaluator assessed the studies independently. The demographic data were blinded for all six evaluators. Repeat examinations were performed 1 month later with the cases presented in a randomly different order, for a total of two assessments per evaluator. The repeat evaluation allowed an assessment of intraobserver reliability. The distribution of the included patients into the different stages of the modified KL classification according to the ratings of the evaluators is presented in the supplementary material.

### Statistical analysis

Continuous variables are presented as mean and median, qualitative variables as percentages and frequencies. To estimate the interobserver and intraobserver agreement, we used the weighted Krippendorff's *⍺* coefficient with simple ordinal weights. Conventionally, an *⍺* of 0 indicates poor agreement; 0.1 to 0.2 indicates low agreement; 0.21 to 0.4 indicates reasonable agreement; 0.41 to 0.6 indicates moderate agreement; 0.61 to 0.8 indicates substantial agreement; and 0.81 to 1 indicates nearly perfect agreement [16]. For each agreement estimate, 95% confidence intervals (CI) were estimated based on conditional standard errors. Statistical analysis was performed using STATA v14® software.

## RESULTS

**Figure 3:**
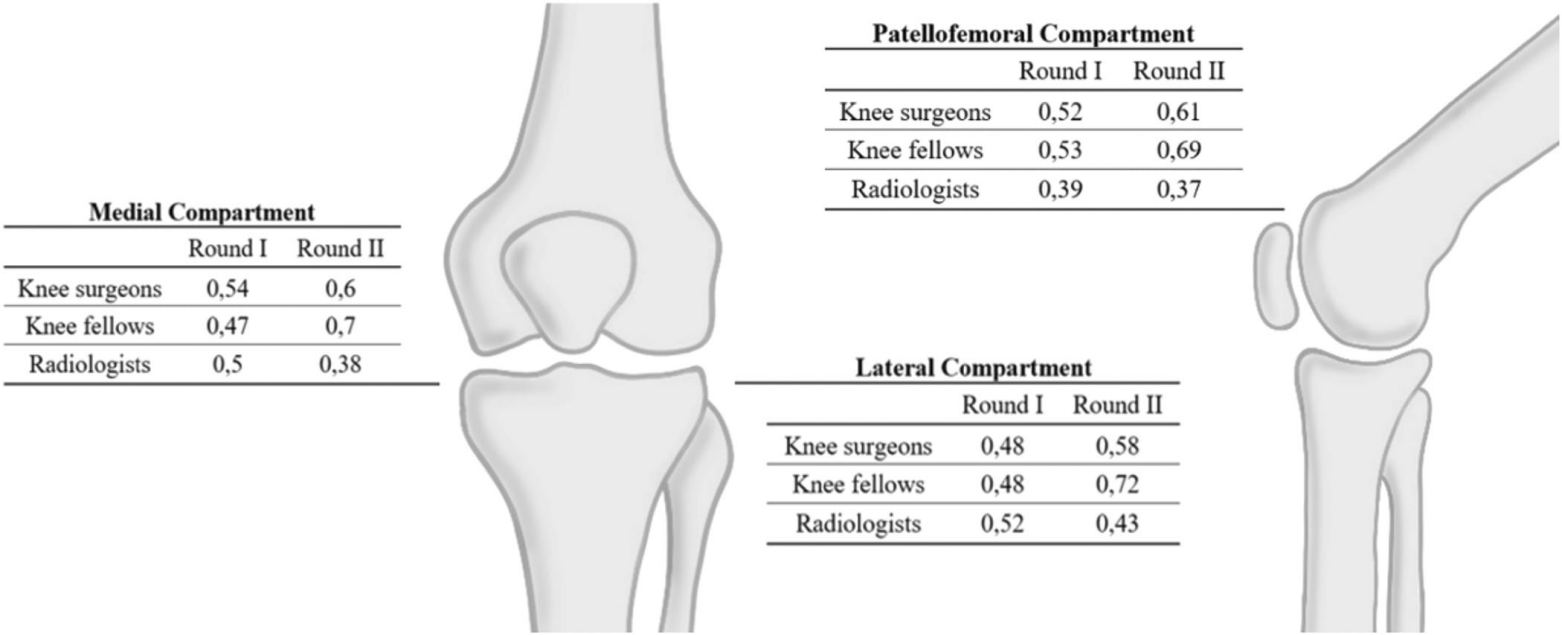
Round I and II of Interobserver reliability Krippendorff's *⍺* coefficient per compartment.

Two hundred and ten knees were included for final evaluation and analyses (53% left knees; 65% females; mean age 56 years old) (Table 3). In the first round of evaluation, there was a moderate agreement between the observers for the modified KL classification in the three knee compartments (Krippendorff's *⍺* 0.48 [95% CI, 0.40–0.55] for the MTFC, 0.46 [95% CI, 0.37–0.52] for the LTFC, and 0.51 [95% CI, 0.44–0.58] for the PFC). In the second round of evaluation, the agreement between observers slightly improved for all the compartments as compared with the first round; the agreement for the PTFC improved from moderate to substantial, whereas for the MTFC and LTFC remained as moderate (Krippendorff's *⍺* value 0.54 [95% CI, 0.47–0.63] for the MTFC, 0.56 [95% CI, 0.48–0.62] for the LTFC and 0.61 [95% CI, 0.54–0.68] for the PFC) (Figure 3).

**Table 3:** Patient characteristics (*N* = 210 knees).

Age (years)^a^	56 ± 20.4
Females, *n* (%)	137 (65%)
Knee laterality, *n* (%)	
Left	111 (52.8%)
Right	99 (47.1%)
Distribution of Kellgren and Lawrence modified classification (%)	
MTFC	
Grade 0	12%–16%
Grade 1	40%–44%
Grade 2	25%–27%
Grade 3	10%–12%
Grade 4	4%–5%
LTFC	
Grade 0	25%–30%
Grade 1	40%–46%
Grade 2	16%–20%
Grade 3	6%–13%
Grade 4	1%–2%
PFC	
Grade 0	12%–30%
Grade 1	29%–31%
Grade 2	21%–26%
Grade 3	9%–20%
Grade 4	6%–9%

Abbreviations: LTFC, lateral tibiofemoral compartment; MTFC, medial tibiofemoral compartment; PTFC, patello femoral compartment.

a The value are given as the mean standard deviation.

**Figure 4:**
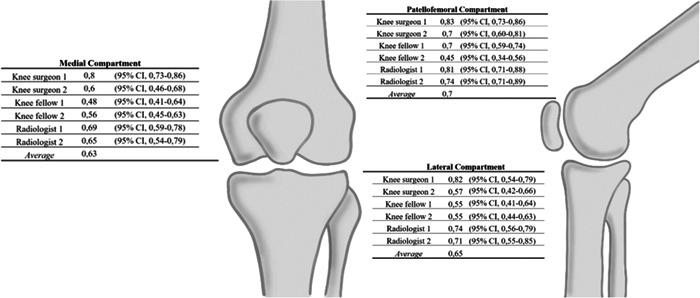
Intraobserver reliability Krippendorff's *⍺* coefficient.

The intraobserver reliability for the three knee compartments ranged from moderate to almost perfect among all the evaluators (0.44–0.83). There was a substantial intraobserver agreement for the three compartments, with the highest average intraobserver reliability achieved for the PFC, followed by the LTFC and the MTFC (Krippendorff's *⍺* 0.7, 0.65, and 0.63, respectively). When discriminating by examiner, the average intraobserver reliability among knee surgeons and radiologists ranged from substantial to almost perfect (Krippendorff's *⍺* 0.81 for knee surgeon #1, 0.62 for knee surgeon #2, 0.74 for radiologist #1 and 0.7 for radiologist #2). Knee fellows had a moderate average intraobserver agreement which was lower than that observed among knee surgeons and radiologists (0.57 for knee fellow #1 and 0.51 for knee fellow #2) (Figure 4).

## DISCUSSION

We propose a modification to the original KL classification that includes a better description of the evaluation parameters and a compartment‐specific approach that separately assess the osteoarthritic involvement in each compartment using AP, lateral and skyline axial‐projection radiographs. Our study found that the proposed modification to the KL classification is reliable among different observers for staging OA in every knee compartment with agreement coefficients that ranged from moderate to substantial. Similarly, this modification showed adequate intraobserver reliability with coefficients that ranged from moderate to almost perfect depending on the experience of evaluators.

The original KL classification scheme for knee OA was first introduced in 1957 [13]. Since then, this classification has been the most widely used to guide clinical decision‐making in the setting of knee OA. Despite the routine application of the KL classification, several authors have noted many limitations of this classification including a lack of recognition of the PFC, the assumption of a linear radiographic progression of OA, and a limited uniformity in its use with the resulting low reproducibility [9].

Although the original report showed a “significant” interobserver and intraobserver agreement for the KL classification [13], subsequent studies show conflicting results with varying levels of interobserver and intraobserver reliability [10, 17, 18, 19, 20, 21, 22] (Table 4). One of the reasons for this discrepancy among studies is the variability in the descriptions of the original KL classification. In a concise report, Schiphof et al. identified no less than five different descriptions of the KL classification in use in the literature [12]. These same authors [20] in a further study evaluated the impact of four different descriptions, or alternatives of the KL classification, on the interobserver agreement and the way knee OA is classified. They showed that most of the alternatives yielded more OA cases than the original KL description and the agreement was significantly affected by the description used.

**Table 4:** Summary of the interobserver reliability of KL classification in several studies.

Author	Coefficient	Result
Wing et al.	ICC	0.85
Günther et al.	ICC	0.81
Köse et al.	Kappa	0.47
Rodriguez et al.	Kappa	0.45
Schiphof et al.	Kappa	0.41
Wright et al.	ICC	0.38
Goncalves et al.	Alpha	0.33

Abbreviations: Alpha, Krippendorff's *⍺* coefficient; ICC, intraclass correlation coefficient; Kappa, Cohen's *κ* coefficient.

The results of our study cannot be directly compared with previous studies due to certain methodological and conceptual aspects such as the included population, the coefficient used to estimate agreement (i.e. Krippendorff's *⍺*), and the fact that our classification is compartment‐based and thus it evaluates agreement per compartment and not globally. However, we found interobserver and intraobserver agreement coefficients that ranged from moderate to almost perfect which might be arguably better than those previously reported for the original KL classification and its variants.

An interesting finding of this study was a higher interobserver agreement for the PFC than for the MTFC and LTFC. The reason for this finding is unknown, however, we suggest that a potential reason for this is the possibility to properly evaluate the PTC in two different projections (lateral and skyline view) as compared with the tibiofemoral (TF) compartments that only can be properly evaluated in the AP view due to the overlap that occurs from these compartments in the lateral projection. Another interesting finding was a higher interobserver agreement in the second versus the first round of evaluation. This may be due to the repetitive use of the classification system by the evaluators which can lead to a better understanding of the scoring system. This finding suggests that clinicians who decide to implement this classification system into their practices may expect this same trend of improvement in the reliability of their ratings with continued use of the classification.

The association between the examiners' training level and the reliability of the KL system evaluations is scarce in the literature [18, 23]. In our study, knee surgeons and radiologists had better intraobserver agreement coefficients than those observed among knee fellows, suggesting that experience is associated with a higher reliability in this classification system. This finding is supported by Wing et al. [21] who showed that a lower limb arthroplasty surgeon and an orthopedic registrar had better intraobserver reliability as compared with an orthopedic intern and medical student when staging knee OA using the KL classification. Further research is necessary to determine the validity and relevance of this association.

An essential part of the proposed modification to the original KL classification is the compartment‐specific staging score. We consider that a separate evaluation of the knee compartments is pertinent, and its rationale is based on an improved knowledge of the natural course and diverse clinical patterns of knee OA. Several authors have studied the natural course and progression of knee OA in each compartment and have suggested distinct patterns of knee OA [24, 25, 26, 27]. In subjects participating in the Cohort Hip and Cohort Knee study (CHECK), Lankhorst et al. found that individuals with early symptoms of knee OA (pain or stiffness) were diagnosed with isolated patellofemoral OA (PFOA) and non with isolated tibiofemoral OA (TFOA). Later, at 2‐ and 5‐year follow‐up, half and two‐thirds of them, respectively, developed combined three‐compartment knee OA [24]. The investigators concluded that combined OA may start in the PF joint and then progress to combined OA [24]. In contrast, other studies [25, 27] have found that the most common pattern of knee OA was the isolated medial TFOA followed by isolated PFOA and/or combined OA. Differences between these studies may be due to demographic characteristics such as varus and valgus alignment and the mechanical axis of the lower limb, which varies between populations [27]. These findings support that knee OA is variable and may have different compromise in each compartment depending on the stage resulting in distinct knee OA patterns. Therefore, a compartment‐specific knee OA classification system is crucial to differentiate between distinct patterns of knee OA, better assess the compromise in each compartment in individual patients and evaluate their progression over time. In addition, a compartment‐based classification may be useful to improve the selection of patients undergoing unicompartmental knee arthroplasty and the comparisons of studies reporting outcomes following this procedure [27]. One potential drawback of using a compartment‐specific classification is the negative impact on the reliability of the ratings. However, this study demonstrates that a compartment‐specific classification system for knee OA can be as reproducible as a classification system with an overall score.

Strengths of this study include the inclusion of a wide spectrum of knee OA patterns and evaluators with varied degrees of experience, and the analysis of agreement using Krippendorff's *⍺* coefficient which is not affected by the known limitations of the *κ* coefficient and its paradoxes [16]. However, the findings of this study should be interpreted after considering the following limitations. First, the observers did not evaluate the cases with the original KL classification, which would make a stricter comparison to the modified KL classification in our analysis. However, previous awareness by the observers of the modified scale could have biased the evaluation of the original classification and vice versa. Second, our study design only evaluated the reliability and not the validity of the modified KL classification. While the substantial data set of over 1000 observations across 210 cases by six observers enhances the robustness and reliability of our findings, a recognized limitation is the omission of a priori and post‐hoc power analyses, due to the intrinsic complexities and interpretative dilemmas involved in implementing these analyses within agreement studies utilizing weighted Krippendorff's *⍺* on ordinal data. Lastly, part of the evaluators included in our study developed the modification and have been familiar with it throughout several years and this may result in an overly optimistic reliability, limiting the generalizability of our results. The evaluation of this modified classification in examiners with different training levels could be a potential target in future studies.

## CONCLUSION

A modified compartment‐specific KL classification enables more practical and detailed description of knee OA involvement and demonstrates acceptable interobserver and intraobserver reliability.

## AUTHOR CONTRIBUTIONS

**Diego Alarcón Perico**: Conceptualization, study design, data collection and analysis, manuscript writing, and final approval of the paper. **Abelardo Camacho Uribe**: Data collection, literature review. **Sara Jaimes Niño**: Data collection, literature review. **María Camila Peñaloza Mayorga**: Manuscript writing, critical revision of the manuscript and final approval of the paper. **Christian Sundfeld**: Data colecction, preparation of research protocol. **Jorge Rojas Lievano**: Data analysis, statistical analysis, data interpretation, manuscript writing, critical revision of the manuscript and final approval of the paper. **Cristal Castellanos Mendoza**: Data collection, literature review. **Rafael Gómez Ramirez**: Conceptualization, data collection and analysis and X‐ray review. **Oscar Rivero Rapalino**: Conceptualization, data collection and analysis and X‐ray review. **Gamal Zayed**: Conceptualization, study design, data interpretation, critical revision of the manuscript, and final approval of the paper. **German Carrillo Arango**: Conceptualization, study design, data interpretation, critical revision of the manuscript, and final approval of the paper. **Klaus Mieth**: Study supervision, project administration, study design, data interpretation, critical revision of the manuscript, and final approval of the paper.

## CONFLICT OF INTEREST STATEMENT

Each author certifies that he has no commercial associations (e.g., consultancies, stock ownership, equity interest, patent/licensing arrangements) that might pose a conflict of interest in connection with the submitted article.

## ETHICS STATEMENT

This study was approved under the code: CCEI‐8601‐2017 by the institutional ethics committee.
